# Experiences of women who reported sexual assault at a provincial hospital, South Africa

**DOI:** 10.4102/curationis.v39i1.1668

**Published:** 2016-11-16

**Authors:** Jeanette M. Sebaeng, Mashudu Davhana-Maselesele, Eva Manyedi

**Affiliations:** 1 Department of Nursing, North West University, South Africa

## Abstract

**Background:**

Sexual assault poses a serious health problem to both the survivor and the health system. Experiencing sexual assault requires women to seek medical and psychological assistance as part of their journey towards recovery. This study examined the experiences of women who received post-sexual assault services from a specialised care centre within a provincial hospital.

**Methods:**

A qualitative, exploratory and contextual design was used to explore and describe experiences of women. Data were obtained through individual in-depth interviews from a total of 18 women aged between 18 and 55 years. Interviews were supplemented by the researcher’s field notes and audiotape recordings.

**Results:**

Findings yielded two main themes: Women expressed their lived experiences of sexual assault characterised by different forms of trauma. The second theme was an expression of a need for safety and support.

**Conclusion:**

Women who experience sexual assault are left with devastating effects such as physical and psychological harm and social victimisation. There is also a need for safety and support towards the recovery of these women. This study recommends that professional practitioners involved in the management of sexual assault be sensitised regarding the ordeal experienced by women and stop perceiving survivors as crime scene ‘clients’ from whom only medico-legal evidence has to be collected. Professional practitioners and family members must be supportive, non-judgemental and considerate of the dignity of survivors. The establishment of sexual assault response teams (SART) is also recommended. There should also be inter-professional education for better coordination of services rendered to sexually assaulted women.

## Introduction

Women who have experienced sexual assault are often subjected to multiple negative outcomes such as physical and mental health problems. The ultimate result of exposure to sexual assault poses the need for women to seek medical and psychological assistance for recovery which becomes a public health problem as purported by Mason and Lodrick (2013). South Africa has the highest rate of sexual assault and was once dubbed the rape capital in the world (Jewkes, Sikweyiya, Dunkle & Morrel 2015). Women of all ages are unfortunately affected by this crime. The South African Police Service provides statistics on sexual assault annually. However, the available statistics are reported as representing the tip of an iceberg as many sexual assault cases are still underreported and under-prosecuted (Jewkes & Abrahams 2002).

Management of sexual assault survivors across the country occurs in the primary health centres and emergency departments of public hospitals. In other hospitals, there are designated centres referred to as the Thuthuzela Care Centres (TCCs) that serve as ‘one-stop stations’ for comprehensive management of sexual assault victims. There are approximately 51 TCCs distributed across the country (Vetten 2015). Survivors must report to the centre within 72 hours following sexual assault for collection of medico-legal evidence, counselling and provision of post-exposure prophylaxis (PEP) which includes prevention of pregnancy, sexually transmitted infections and HIV.

### Problem statement

The magnitude of the prevalence of sexual assault in the North West Province for the year 2009–2010 was reported to be 5295 (BuaNews 2009). Although these numbers are likely to significantly underestimate the true incidence of sexual assault, they provide the basis for the acknowledgement of the existence of the crime in the province. It is evident that not much has been done by previous studies to formally explore and document the experiences of sexually assaulted women in the province. Hence, the aim of this study was to explore and describe the experiences of sexually assaulted women who received care at a TCC in a hospital within the North West Province.

### Definition of concepts

**Sexual assault** – A person who unlawfully and intentionally sexually violates a complainant without the consent of the complainant is guilty of the offence of sexual assault according to the Sexual Offences Act 32 of 2007.

**Experience** involves personal knowledge, personal involvement and first-hand knowledge and exposure (Myburgh & Poggenpoel 2009:448).

**Victim** – Any person alleging that a sexual offence has been perpetrated against him or her (sexual offences and related matters).

## Research method and design

The study took place in one of the TCCs within a provincial hospital in South Africa. A qualitative, phenomenological, exploratory and descriptive design was used which is contextual in nature. The target population included women who had experienced sexual assault and had reported at a provincial hospital for management. A non-probability purposive sampling was used targeting participants aged between 18 and 55 years. The participants were purposively selected as they were deemed relevant for answering the research questions posed. Self-determination of participants was ensured by sampling participants who were legally and psychologically legible to consent. Participation was voluntary and participants were given an alternative of having interviews conducted at the centre or at their respective homes, depending on the time and place convenient to them. Demographic data were not included in this study as they were regarded as identifiers with the possibility of linking participants, thereby breaching the anonymity and confidentiality agreed upon. Data were collected through in-depth face-to-face interviews with the use of audiotapes for capturing voices. One central, open-ended question was posed to each participant as follows: What is your experience regarding the sexual assault incident that happened to you? The researcher took field notes supplemented by audiotapes. Interviews took one and a half to two hours per participant. The emotions of the participants were kept in check given the sensitivity and private nature of the topic. Interviews were paused for those participants who became emotionally provoked during the process and were provided with water and facial tissues. The interview was continued after the participant had calmed down. Verbatim transcriptions from audiotapes were used as primary data source supplemented by the researcher’s field notes. Data were independently analysed by the researcher through an open-coding method using Tesch’s steps of data analysis (Creswell 2009).

## Ethical considerations

The study was commenced after obtaining clearance from the ethics committee of the North West University, permission to access participants from the North West Provincial Department of Health and permission from the Mafikeng Provincial Hospital chief executive officer and the deputy director nursing. Participants were further consulted on a one-on-one basis providing them with detailed information regarding the purpose and procedures to be entailed in the study. Basic ethical principles applicable to research performed on human subjects such as respect for persons, beneficence, justice, confidentiality, anonymity and right to privacy were all observed.

## Trustworthiness

The study applied Guba’s four basic principles, namely credibility, dependability, transferability and confirmability as discussed in Polit and Beck (2008) to ensure trustworthiness.

Measures to ensure credibility of the study such as using qualitative phenomenological design relevant for exploring and describing human lived experiences, purposive sampling of participants deemed informative regarding the questions the study seeks to answer and prolonged engagement was employed. Data were also captured verbatim as field notes and by using audiotape recordings to allow validation later when out of the field. Dependability was achieved through keeping audit trails. Sufficient description of participants’ experiences and the methods used were aimed at enabling the reader who wishes to apply the findings to other settings, thus achieving the transferability principle. Data collected were coded and recoded at different intervals by the researcher and validation of the results was done using the co-coder. To achieve confirmability, clear and transparent reporting of the research process and findings of the study was ensured. An independent co-coder was also involved for verification of the findings. Two main themes were generated from data analysis. See Table 1.

**TABLE 1:** Summary of findings.

Theme	Categories	Subcategories
Participants shared their lived experiences regarding sexual assault characterised by different forms of trauma	Physical trauma	Injuries sustained owing to physical assault Pain owing to forced penetration from multiple perpetrators
	Emotional/psychological trauma	Sleeplessness Fear Isolation/withdrawal
	Interpersonal relationships with opposite gender	Anger towards men
Participants expressed their need for safety and support	Views related to support received	Thuthuzela Care Centre Police Services Support from family and friends
	Views related to safety and insecurity	Threats Insecurity

*Source*: Authors’ own work

## Victims shared their lived experiences regarding sexual assault characterised by different forms of trauma

The first theme had three categories, namely physical, emotional/psychological as well as social trauma which had further subcategories:

### Physical trauma

Some participants reported to have been deliberately subjected to physical assault by the perpetrators by means of weapons, fists and choking to gain their cooperation.

#### Injuries sustained owing to physical injuries

Participant 1 explained her ordeal as follows:

‘When he realized that I am fighting back, he grabbed me by the neck and throttled me. So, I don’t know as to whether I ended up having a blackout because when I woke up, I was lying on the ground on my back and he was already on top shouting: Hey! What’s wrong with you, don’t you see that I want to ejaculate? I tried fighting him again and he grabbed and held my hands down, forced himself on me and finished.’

Participant 2 said:

‘I was raped by two unknown guys. They used knives and sticks to attack me. They then both raped me. I sustained a cut here and here [*pointing at different sites on the head and the ear*]. I was sutured on the head but they did not suture the ear and now it looks ugly.

[*Two suture lines were visible at different sites on the scalp and the lower left ear was divided into two pieces as a result of being cut*.]’

Participant 3 expressed herself as follows:

‘He closed my throat with his hands. He was having a press button knife, giving me orders using his knife but he did not stab me. He only hit me on the head and it was swollen. He raped me for the first and second time. Now when he tried for the third time, I managed to escape. I ran and get through a barbed fence to my neighbours and got scratched on both legs. I was having cuts all over. I got through the door of my neighbours holding my panty in my hand. I was bleeding and my night dress was soaked in blood.’

Participants in this study sustained physical injuries ranging from bruises, lacerations to stab wounds. The most common form of assault used by perpetrators was choking or holding the victims by the neck. Those who tried to fight their perpetrators were overpowered and were subjected to forceful sexual penetration as well as use of physical power which led to physical injuries. There were physical injuries in 61% of the cases of which 83% were on the head/face, neck, torso/abdomen and extremities (Maguire, Goodall & Moore 2009).

#### Pain from forced penetration by multiple perpetrators

Participants were subjected to genital injuries and painful intercourse owing to forceful penetration by multiple perpetrators. Participant 4 explained as follows:

‘I was about to ascend the bridge when four guys approached me. One plugged my mouth and the other two held my hands. I was wearing white pants and they pulled them off. One guy said: I will be the first, I am going first, [*started crying*]. He started raping me and the other one said to him: hey man, are you not done yet? They were not even using condoms [*becoming more agitated*]. I was terrified and was praying God to save me, to help me survive this. After finishing, the other one came. After they have all finished, I put on my clothes. The other one suggested that they kill me to prevent me from reporting them to the police and his friends disagreed with his idea.’

Participant 6 explained her ordeal like this:

‘We were asleep and I suddenly heard a lot of noise, people talking loud and breaking into our house. We [*my younger sister and I*] then fled into our dad’s room who is very old and weak. They entered into my dad’s room, took the bed mattress off and onto the floor. They threw me on the mattress and started raping me. It was a group of people, about nine …, the whole group raped me. When the other finished, the other one would follow, just like that.’

Engaging in a sexual intercourse activity according to McQuid-Mason *et al.* (2002) is a process which requires normal human physiological response that occurs in four phases. The first phase is the excitement phase that prepares the female genital tract to receive the penis by increasing lubricating secretions and muscle relaxation. The researcher believes that participants suffered pain and genital injuries given that these mentioned phases of sexual intercourse were not followed in their case. Moreover, Anderson *et al*. (2009) state that whether a woman has consensual or non-consensual sex, she still suffers genital injuries. The fact that women in consensual sex are subjected to genital injuries led the researcher to conclude that participants in this study sustained more genital injuries as they were involved in non-consensual forceful multiple perpetrator intercourse.

### Impact of psychological trauma

Participants experienced varied psychological trauma following sexual assault.

#### Sleeplessness

Sleep problems were found to be more prevalent in most sexually assaulted women.

Some sleep disturbances or interruptions following sexual assault were expressed by participant 14 as follows:

At night I will wake up. It will be like someone is walking in the house. I will wake up and knock on my children’s rooms. It will be like someone is entering the house. It took me some time to accept that this thing is not real. Since then, I don’t even go out at night, I am scared.’

Participant 8 said:

‘Hey, this rape has troubled me a lot, I don’t sleep. At times I just wish that it becomes daylight for good without the night setting in.’

Steine *et al*. (2011) reported that sleeplessness is more common among rape victims. Most frequently reported symptoms included nightmare-related distress, sleep paralysis, nightly awakenings, restless sleep and tiredness as reported by participants in this study.

#### Fear

It was also discovered that participants experience fears resulting from the experience of sexual assault. This is how participant 12 expressed herself:

‘So, since after this incident … I am still having this fear. I am still scared. I am asking myself what will happen should I meet them [*perpetrators*] on my way.’

Campbell *et al*. (2009b) confirmed that fears and phobias are common psychological defences. Some survivors may develop fear of crowds, of being alone, of having sex or they may feel a general paranoia and anxiety (Culbertson *et al*. 2001; Mezey 1997).

During the process of dealing with the sexual assault incident, this is what was said by participant 7:

‘At first I thought of killing myself. Like I was telling myself …, we are five girls at home neh, and this thing only happen to me among my mother’s children … from five …, I asked myself the reason for living eintlik [*in fact*]?’

Studies have shown a link between rape or sexual assault and suicide attempts (Maguire *et al*. 2009; Ullman 2004; Vickerman & Mangolin 2009).

#### Isolation/withdrawal

Participants further expressed their feelings with regard to interaction with other people post-sexual assault. Participant 16 expressed a feeling of social isolation and loneliness as follows:

‘Sometimes I feel like I can just be alone in the house, lock myself in the house where I won’t be seen by anyone.’

## Interpersonal relationships with opposite gender

### Anger towards men

After having been sexually assaulted, some women started experiencing different feelings towards men. Participant 9 expressed herself as follows.

‘I want nothing to do with men. When I see a male person, I start thinking about or remembering those things.’

Participant 3 said:

‘I don’t feel comfortable, especially around guys. I told my friend that I hate guys. For as long as you are a man, I hate you. I told her that this thing that has happened has made me to hate all of them. I hate them because even a person you think can never do this thing is going to do it also.’

Participant 10 had this to say:

‘This thing is troubling me. I don’t have feelings for males anymore. When I see a male person, I start thinking about or remembering those things.’

From the findings, it is evident that sexual assault impacted negatively on the social functioning of participants as they have developed different perceptions about men compared with before the assault. Many victims of rape experience temporary impairment of social functioning, particularly in social withdrawal and avoidance as well as restriction of former interests and activities (Mezey 1997).

Participants expressed the need for support and safety.

### Participants shared varied experiences related to services received and their need for safety and support

#### Views related to support received

This section discusses supportive and non-supportive behaviours as experienced by participants from health professionals at the TCC, the police and family members.

#### Thuthuzela Care Centre

Findings on service provision revealed that health professionals displayed both positive and negative attitudes while rendering care to participants. However, most participants in this study were satisfied and reported feeling better. Participant 17 said:

‘Ja well, I felt ok after speaking to them [*health professionals*]. Even the story about this incident …, I don’t think about it a lot unless receiving a phone saying I must come to the hospital about the rape issue like now. It is then that my mind starts thinking of it. So far, they have done everything.’

Participant 15 said:

‘They helped me as they could. The doctor came and did his work and after that I bathed. They gave me a clean panty. I was then seen by a social worker and policewoman. They gave me some food. It was bread with peanut butter and tea. At least people there were happy for me and I was treated well.’

Participant 14 indicated dissatisfaction with the behaviour of some personnel:

‘One thing that made me sick was going home just like that without bathing after being raped by four men. You see going like that with the police without taking a bath …, I wasn’t given a chance to shower. I wasn’t asking too much …, at least a bath. I smelled bad.’

It is evident from the narrations that positive support provided to sexual assault survivors made a difference in their lives while negativity after their traumatic experience added more pain. According to Chivers-Wilson (2006), feelings of anxiety or depression may be even stronger and more harmful if the survivor does not receive support from their family, friends or professionals.

#### Police services

Participants stated that reporting to the police was significant to them as they felt they needed to take action against their perpetrators.

Participant 11 narrated:

‘There were two policemen and a policewoman. So, this lady was like …, she was not interested niks [*not at all*] in the case. It was issues of ’hey man, let us … we will see that person; hey we are wasting time. Heh! This …, this and that’. So, I was asking myself, because she is a woman, she is supposed to at least be more attentive and sympathetic in this case, but she was the one who was saying hey, let us go. We won’t get this person, this case is difficult, such things.’

Same participant continued:

‘So, when I realized that the police are dragging their feet, I told myself that you know what, its fine, it has happened, I was raped. One thing that I have to do is just to move on with my life. Since then, they never said anything, since last month. They are not saying anything.’

Participant 7 of whom the perpetrator was not arrested said:

‘I feel disappointed on the police to be honest. These people [*perpetrators*] are there and they don’t stay in one place, you understand. Today they are there, the following day there, next week there and people are reporting and there is nothing happening.’

Most participants were not happy with the way police responded or acted to their victimisation. Some participants gave up on their cases as a result of the passive reactions displayed by police officers. Felson and Pare (2008) state attitudes and behaviours of the agents of the criminal justice system often are harmful and demoralising to victims and can cause women a deeper despair than the abuse itself. These negative reactions include reactions that are unresponsive to the victims’ needs or may even be overtly harmful (Borja, Callahan & Long 2006). These behaviours have been termed secondary victimisation (Campbell 2009).

#### Support from family members

Participants expressed a need for support from family members, friends and partners. Participant 13 expressed lack of support in this manner:

‘I don’t have a mother or a father, you understand. I stay with my aunt and we live with her husband. You know sometimes you wish to discuss these things with a female person, so there is no one to speak to. My aunt and her husband are always sitting together and at times I feel like I am disturbing them when I talk about this issue. My aunt is at times too quiet.’

Participant 16 said:

‘When I spend some time with my friends and my boyfriend, I feel okay, but sometimes when I am alone, maybe reading a book or something, and I recall this rape story, my heart starts pounding. I experience some stress, I feel like talking to someone. Ae, I don’t want to recall it.’

This is what participant 8 had to say:

‘I used to hear stories about rape you know. I never thought it would happen to me. It scared me. I was tortured and now am looked at by my own community members like I am a piece of trash. It is like I had a choice. My neighbours here you know …, has spread this thing [*sexual assault*] around the whole village. They are talking about it everywhere.’

Participant 15 said:

‘If only there was someone who can tell me that I am not the only one … that it will be okay sometime.’

Opening up and talking about the incident of sexual assault and just being listened to were considered important by the participants. Positive reactions from family and friends as informal support providers are associated with benefits in the aftermath of trauma (Campbell *et al*. 2009a). Living with family and feeling close to family members are related to better adjustment. Having a strong social support network could decrease the likelihood of victims experiencing negative sequelae following sexual assault. This finding was affirmed by Campbell *et al*.’s (2009a) ecological model of the impact of sexual assault on women’s mental health. The microsystem factor of this model posits social support from family, friends and intimate partners as facilitating sexual assault recovery.

### Views related to safety and insecurity

Participants related their stories in relation to how they were threatened and degraded by perpetrators.

#### Threats

Participants were exposed to various forms of threats from their perpetrators. These threats ranged from swearing, threatening to kill and name calling.

Participant 4 said:

‘I screamed. They plugged my mouth and said: “voetsek you are making noise …!” They dragged me to a secluded area and then said “take off your clothes otherwise we are killing you [*crying*]”. I said to them don’t kill me please, do what you want but please don’t kill me.’

Participant 9 related her story in this manner:

‘And then he said eish, today you know my blood is boiling. I feel like killing a person. I don’t know whether I am going to kill you or what. I started pleading with him. I was scared when he told me that he wants to kill me, because he was having a long knife.’

Participant 10 said:

‘He knocked on the window and said: open, I know you are crippled and I know that you stay alone. There is nothing that you can do for yourself. Open so that I rape and kill you inside that house. I went out through the other window and I met him outside the house. We started fighting. I tried to scream then he threatened me with a knife, I kept quiet. I got raped behind my house [*looking down on the ground with tears on her face*].’

The same participant continues:

‘He was calling me all sorts of bad names’. [*Tears coming down her cheeks*] ‘He told me that he wants my koek [*vagina*] and that he wants it anally too’.

Participants in this study were abused verbally, threatened to be killed or called degrading names. As a result, they gave up and were forced into sexual activities against their will. According to Avengo *et al*. (2009), threats of force, either physical (fists or choking, knife, gun or other means) commonly occur during sexual assault.

#### Insecurity

Participants shared their experiences of their houses being broken into and being sexually assaulted. These experiences shattered their assumptions about homes being safe and protected places.

Participant 12 said:

‘I no longer want to stay there, it is not safe. I wish that we get ourselves a place you know. You see my other siblings are scared to come here because of this incident. My younger sisters and even my mother doesn’t want to come here in … she is also scared.’

Participants reported that their privacy was invaded as they were attacked in their own homes while sleeping when they least expected it. Other participants no longer trusted the safety of their homes and moved out. For most women, the sexual assault and its aftermath shattered previously held assumptions about safety and trust (Mezey 1997).

## Conclusions

Sexual assault has devastating effects on women and it affects both their physical and emotional health. If not properly managed, victims of sexual assault may end up not recovering from the traumatic ordeal they have experienced. In order to assist them towards recovery, social systems taking care of victims must be well-sensitised, non-judgemental and have the ability to treat victims with dignity and respect. Support towards recovery following sexual assault is needed from both formal and informal systems. If victims receive services they need and are treated empathetically, their recovery may be facilitated (Campbell *et al*. 2009a). It is also evident from the findings that support from the family, friends and partners played an important role in the recovery process of victims of sexual assault.

## Recommendations

This study recommends the formation of focused sexual assault response teams (SART) that should be able to provide required comprehensive care to the victims of sexual assault. Mason and Lodrick (2013) suggested that people interacting with sexual assault victims understand the physical and psychological consequences and their long-lasting effects on victims’ well-being and future functioning. An inter-professional education (IPE) is also recommended to enable caregivers to learn together about sexual assault management in order to provide coordinated services. The South African Police Services (SAPS) must also come up with inclusive functional community programmes that aim at ensuring safety and security of inhabitants.
